# Examining Technology Perspectives of Older Adults With Mild Cognitive Impairment: Scoping Review

**DOI:** 10.2196/78229

**Published:** 2025-10-30

**Authors:** Snezna Bizilj Schmidt, Stephen Isbel, Blooma John, Ramanathan Subramanian, Nathan Martin D'Cunha

**Affiliations:** 1 Faculty of Science and Technology, Centre for Intelligent Computing and Systems (CICS), School of Information Technology and Systems, University of Canberra, 11 Kirinari Street, Bruce, ACT, 2617, Australia, 61 0416377482; 2 Faculty of Health, Discipline of Occupational Therapy and Centre for Ageing Research and Translation, University of Canberra, Bruce, Australia; 3 Faculty of Health, Centre for Ageing Research and Translation, University of Canberra, Bruce, ACT, Australia

**Keywords:** mild cognitive impairment, MCI, technology adoption, multimodal, interaction, preference, usability, user experience, UX

## Abstract

**Background:**

Mild cognitive impairment (MCI) affects up to 20% of people older than the age of 65 years. The global incidence of MCI is increasing, and technology is being explored for early intervention. Theories of technology adoption predict that useful and easy-to-use solutions will have higher rates of adoption; however, these models do not specifically consider older adults with cognitive impairments or the unique human-computer interaction challenges posed by MCI. There are gaps in understanding the combined impacts of aging and cognitive impairment on factors affecting technology adoption for older adults with MCI, and it is not clear how MCI impacts human-computer interaction and device and interaction modality preferences in this population.

**Objective:**

This study aimed to collate perspectives from older adults with MCI about technology solutions proposed for them, to understand whether solutions are perceived as useful, easy to use, and what changes are suggested. It also identifies which devices and interaction modalities are preferred, and other factors that may affect usage and adoption.

**Methods:**

This scoping review was completed according to the PRISMA-ScR (Preferred Reporting Items for Systematic Reviews and Meta-Analyses Extension for Scoping Reviews) guidelines. A consistent search was performed across 9 electronic databases (ACM Digital Library, EBSCOhost CINAHL Plus with Full Text, EBSCOhost Computers and Applied Sciences Complete, Google Scholar, JMIR Publications, IEEE Xplore, EBSCOhost MEDLINE, Scopus, and Web of Science Core Collection) for studies published between January 1, 2014, and May 1, 2024. Extracted data were analyzed using inductive thematic analysis.

**Results:**

We identified 4271 studies, and after the removal of duplicates and screening, 83 studies were included for data extraction. Inductive thematic analysis of feedback from older adults with MCI about technology solutions proposed for them identified five themes: (1) purpose and need, (2) solution design and ease of use, (3) self-impression, (4) lifestyle, and (5) interaction modality. Solutions were perceived as useful, even though gaps in functional support exist; however, they were not perceived as entirely easy to use due to issues related to usability and user experience. Devices that are lightweight, portable, familiar, and have large screens are preferred, as is multimodal interaction—particularly speech, visual or text, and touch.

**Conclusions:**

Using technology can create feelings that positively or negatively affect a user’s comfort, confidence, and overall well-being. Older adults with MCI value independence and autonomy, and solution designs should support these. Usefulness, ease of use, security, privacy, cost, physical comfort, and convenience are important considerations for technology use. Reliable technology creates trust, confidence, and feelings of empowerment. This review recommends future work to (1) improve usability and user experience, (2) enhance personalization, (3) better understand interaction preferences and effectiveness, (4) enable options for multimodal interaction, and (5) more seamlessly integrate solutions into users’ lifestyles.

## Introduction

### Background

Mild cognitive impairment (MCI) affects a person’s memory or how they think, feel, or behave. It is not a normal part of aging, but up to 20% of adults older than the age of 65 years may be affected, and up to 15% of those may progress to dementia [1,2]. Historically, MCI was considered a decline in a person’s memory, but the criteria and definition of MCI have expanded with more research to include other cognitive domain deficits and affective attributes such as language, executive functioning, or visuospatial ability [3-5]. A memory decline is only labeled single-domain amnestic MCI. A decline in 1 nonmemory cognitive domain is labeled single-domain nonamnestic MCI, and a decline in memory and one or more other cognitive domains is labeled multiple-domain amnestic MCI. People with MCI may forget things more frequently, lose items more often, struggle to remember words or have problems with language, have trouble making decisions or following instructions, lose their train of thought, have new difficulties regulating emotions, behave more impulsively, have trouble with visual perception, have trouble sleeping, experience motor coordination issues, or be less able to follow daily routines [2]. Given that visual, perceptual, cognitive, and behavioral functions are needed for human-computer interaction (HCI), the limitations associated with MCI create challenges for HCI, such as difficulties remembering process steps or commands, understanding instructions, interpreting feedback, correcting errors, or maintaining focus and attention. Changes to emotions, feelings, or mood may also make MCI more difficult and thereby create frustration, increase fear of making mistakes, and reduce self-confidence, all of which have been shown to contribute to factors influencing technology adoption (TA). These challenges can be managed through a considered design of technology solutions.

The global prevalence of MCI is increasing and placing additional stress on both health and aged care services [6]. The number of older Australians with MCI is estimated to grow from 884,000 in 2022 to 2.03 million in 2070 [7,8]. The Australian Royal Commission into Aged Care Quality and Safety [9] noted that aged care services are already under pressure and recommended exploring technology options to address the growing needs of older Australians. Australia’s National Dementia Action Plan 2024‐2034 [10] recommended that action be taken to (1) implement evidence-based early interventions to reduce cognitive decline, and (2) provide support and resources for people with MCI.

Research has shown that technology provides opportunities to increase health care service productivity [11], but only if solutions are adopted by the target users. Aging may result in slower cognitive speed, poorer memory, reduced concentration, and negatively impact vision, hearing, speech, dexterity, mobility, and learning abilities. These limitations have been found to discourage the use of technology [12], and studies have shown that the use of digital health solutions is lower among older adults than younger adults [13]. In addition, as people experience MCI uniquely and with different symptoms, older adults with MCI will have different and diverse needs for technology support.

### Literature Review

TA has been studied from several perspectives. Early models, such as the technology acceptance model (TAM) [14], TAM2 [15], and TAM3 [16], take a behavioral perspective and consider TA in an organizational context. They describe TA as being dependent on a user’s perception of the technology’s usefulness, ease of use, and the determinants of these. The unified theory of acceptance and use of technology (UTAUT) [17] considers TA from a combined behavioral, psychological, and information management perspective, and UTAUT2 [18] extends UTAUT to consider TA outside of business settings, such as at home, and shows that the context of use has an impact on the relevance and contribution of determinants. However, these models were not developed based on data about older adults with cognitive impairment or the assessment of health care technologies. More recent models, such as the senior technology acceptance model (STAM) [19] and the model for the adoption of technology by older adults (MATOA) [20], focus on older users and the effects of physical and cognitive aging on TA. They note that ease of use becomes more important as users age, and MATOA identifies self-management (the extent to which a person can remain independent and maintain control of their life, emotions, and role) as a determinant for intention to use. The lifespan theory of control [21] describes the importance of control and states that people will attempt to retain control of how they behave and interact with their external environment for as long as possible because control contributes to a sense of well-being. It is therefore relevant to consider user empowerment as a valuable design feature in technology solutions designed for older adults, and this is supported by a literature review of older adults’ intention to use technologies [22]. STAM and MATOA do not specifically consider TA by older adults with cognitive impairments or technology designed for health care or assistance with activities of daily living. The health care technology acceptance model (H-TAM) [23] describes acceptance of health care technologies by older adults with hypertension. It includes many factors that appear in TAM-based TA models and proposes compatibility (how well the technology integrates into a person’s everyday life) as a determinant of facilitating conditions.

A study of people’s intention to use service robots [24] suggests that TA models may need to be revisited to incorporate the influence of technology innovations such as smart technologies and robots, which may be adaptive and exhibit human-like features. These advances have enabled new uses and new modes of interaction, and users can now choose their device and interaction mode, which is empowering. However, it is not clear how this impacts TA for older users with cognitive impairments, in particular, perceptions of usefulness and ease of use, which are shown to be determinants of TA. Researchers have investigated the effectiveness of unimodal and multimodal interaction for older users and found that modality affects use [12,25-29]; however, these studies did not include older adults with cognitive impairments. Subtle speech differences can be observed in people with MCI [30], and literature reviews have reported on the use of voice-activated technology solutions by older adults [27,31,32]. However, these did not always include older adults with cognitive impairment as participants.

There are gaps in understanding the combined impacts of aging and cognitive impairment on factors affecting TA for older adults with MCI. It is also unclear how MCI impacts HCI and device and interaction mode preferences. This scoping literature review examines published studies about technology solutions developed for older adults with MCI (amnestic or nonamnestic, and single- or multi-domain) to answer the following research questions:

- Research question 1 (RQ1): What are the opinions of older people with MCI about the technology solutions that have been proposed for them? (A) Are they useful? (B) Are they easy to use, or are changes and improvements suggested?

- RQ2: What feedback do older people with MCI provide about usage? (A) Preferred devices? (B) Interaction modality and ways of use?

To our knowledge, this is the first study that collates opinions and feedback from older adults with MCI about technology solutions proposed for them.

## Methods

### Overview

This scoping review design was guided by previous literature [33,34] and is reported according to the PRISMA-ScR (Preferred Reporting Items for Systematic Reviews and Meta-Analyses Extension for Scoping Reviews) guidelines [35]. It was preregistered on the Open Science Framework Storage (United States) [36].

### Eligibility Criteria

An electronic database search was performed to identify studies published in English between January 1, 2014, and May 1, 2024. A 10-year period is considered sufficient given the fast evolution of technology and the potential for change in people’s opinions.

### Information Sources

The search was performed consistently across nine electronic databases (ACM Digital Library, EBSCOhost CINAHL Plus with Full Text, EBSCOhost Computers and Applied Sciences Complete, Google Scholar, JMIR Publications, IEEE Xplore, EBSCOhost MEDLINE, Scopus, and Web of Science Core Collection).

### Search Strategy

The search strategy was developed with the assistance of a qualified librarian. The following search query was used in all databases:

((MCI OR "mild cognitive impairment” OR “mild cognitive disability” OR “mild neurocognitive”)AND(technolog* OR ICT OR smart OR wearable* OR computer OR PC OR laptop OR tablet OR “touch screen” OR “touch-screen” OR “mobile phone” OR “mobile device” OR “personal device” OR robot OR reality OR VR OR “assistive technolog*” OR “embodied conversational agent” OR ECA OR multimedia OR “multi-media”)AND(modalit* OR mode OR channel OR interact* OR engage* OR touch OR type OR voice OR gesture OR use OR usage)AND(preference* OR experience* OR perception* OR attitude* OR feeling OR practices OR “technology acceptance” OR qualitative OR “design science” OR “innovation resistance theory”))

### Selection Criteria

Studies that satisfied the search criteria were imported into the referencing software program EndNote (Clarivate Analytics) and then uploaded to Covidence (Veritas Health Innovation) to remove duplicates and for screening [37].

Three reviewers and the primary author screened the titles and abstracts of each study and excluded any that did not meet the following inclusion criteria:

- Participants have MCI and were living independently in the community (not in residential aged care)
- Participants used or evaluated the technology solutions or devices
- Participants provided feedback about a technology idea, paper prototype, or abstract concept
- The study assessed one or more of the participants’ experiences, feelings, or attitudes regarding the technology, and preferences for use

Papers were excluded if:

- Participants lived in residential care or a nursing home
- The paper did not report on participant feedback.
- The paper reported only on the efficacy of the solution
- If the study included multiple participant cohorts, the feedback was not identifiable or reported by cohort

Where it was unclear if a study met the inclusion criteria, or there was a disagreement among reviewers, the study was carried forward into the full-text review.

The primary author independently reviewed all full-text studies extracted from the previous stage. The remaining authors shared the role of second reviewer. Full-text papers were included if they reported the following:

- Research about participants’ use of technology and their experiences, feelings, attitudes, opinions, or preferences
- If multiple participant cohorts were included, the data were identifiable and reported by cohort
- Participants were assessed for MCI
- Participants had no comorbidities that cause neurological issues or affect cognition

Any disagreements were resolved by discussion among the authors.

### Data Analysis Process

Data were extracted into a structured Microsoft Excel spreadsheet by the first author and evaluated by 2 other authors. Data were extracted about (1) study details (such as author, location of study, year published, journal name, study aim, study type, technology being evaluated, and participant details) and (2) participant feedback. Participant feedback was recorded as any reference to (1) a participant’s opinion about the technology (usefulness, ease of use, usage, and context of use), (2) how the solution made them feel, (3) preferences for device or interaction, (4) issues or challenges with use, and (5) recommendations for changes. Statements were captured as written, relating to the type of technology and solution.

All extracted data were reviewed for familiarization and to start the process of identifying similarities, relationships, and trends. Participant feedback was organized and analyzed using inductive, thematic analysis as described by Thomas and Harden [38], to ensure transparency and traceability from the data to the outcomes. This process has been used effectively to complete other thematic analyses of qualitative data that describe people’s experiences, opinions, and feelings.

Inductive thematic analysis of participant feedback was guided by the 2 research questions and completed in 4 steps:

1. Keyword allocation: each feedback item was assigned one or more keyword phrases that succinctly described its meaning. The first author used existing keywords or created new ones as needed.
2. Consistency check: review of all feedback items with the same keyword to ensure consistent allocation.
3. Category grouping: related keyword phrases were grouped into higher-level categories, which were then named.
4. Theme creation: the categories were reviewed, and 5 abstract themes were created to provide meaning to the feedback and answer the research questions.

During the above 4-step process, there was a continuous feedback cycle with coauthors.

## Results

### Overview

**Figure 1.**
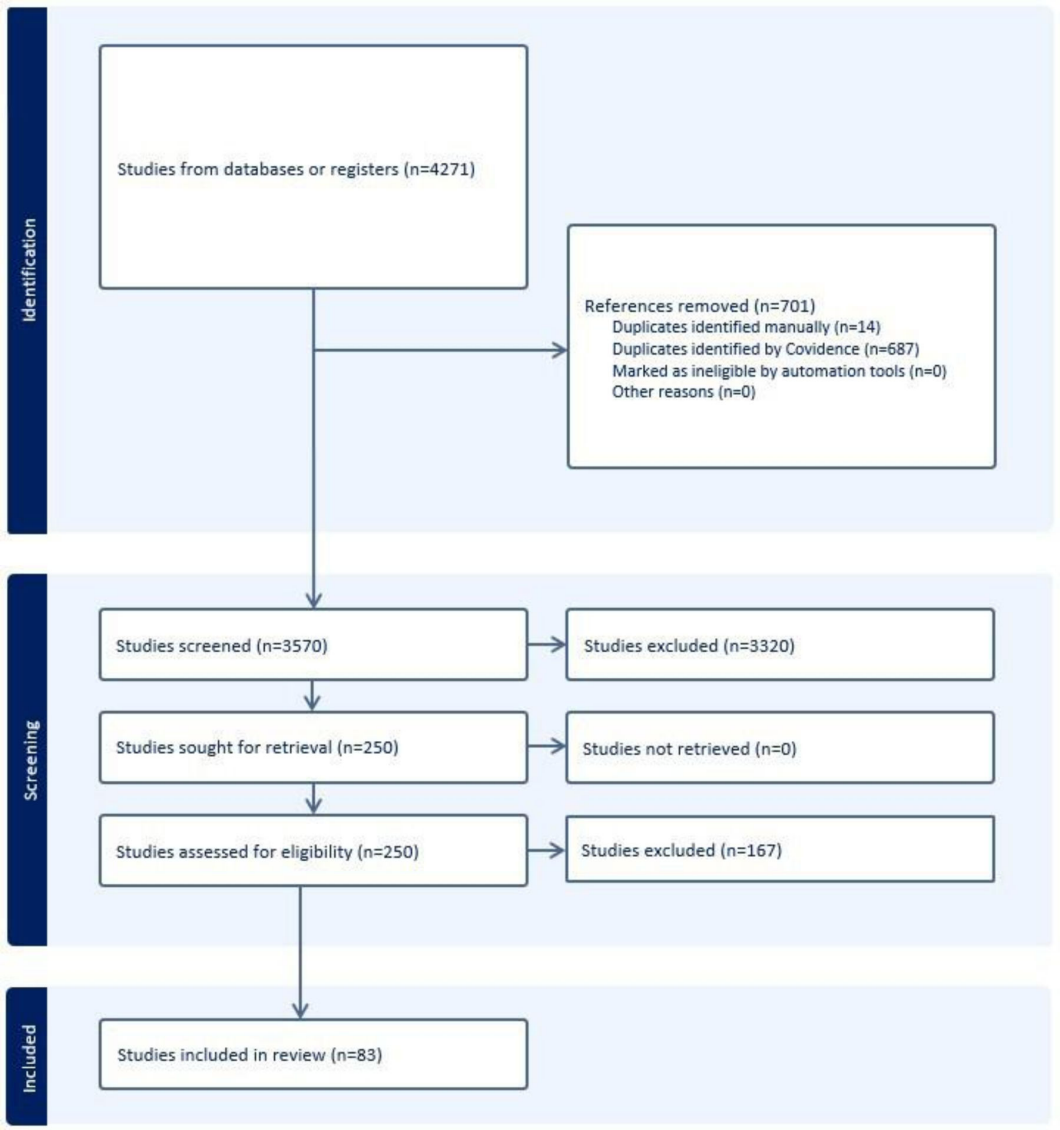
Flow of studies through the PRISMA-ScR (Preferred Reporting Items for Systematic Reviews and Meta-Analyses Extension for Scoping Reviews) process.

A total of 4271 studies were imported for screening, 687 duplicates were identified and removed by Covidence*,* and 14 duplicates were identified and removed manually. A total of 3570 studies were screened by title and abstract, and 3320 studies were subsequently excluded. The full text of 250 studies was assessed for eligibility, and 167 studies were excluded, leaving 83 studies for data extraction. Figure 1 presents the flow of studies through the PRISMA-ScR process, and Table 1 provides a summary of the studies and extracted data, sorted alphabetically by the author.

**Table 1.** Summary of studies, sorted alphabetically by author.

Study details	Study type	Type of technology	Purpose of the solution	Participant details
Afifi et al [39]	Quantitative	VR^a^ headset	Social interaction, messaging, chat, or information provision	Female: 18; male: 3; location: community center; 9 participants had MCI^b^
Bartels et al [40]	Mixed methods	Mobile phone	Lifestyle (ADL^c^) support, social interaction, or companionship	Female: 5; male: 16; location: home
Beentjes et al [41]	Mixed methods	Touch-screen laptop or tablet	Lifestyle (ADL) support, social interaction, or companionship helps people find apps for self-management and meaningful activities	Female: 8 (3 in experimental group and 5 in control group); male: 12 (7 in experimental group and 5 in control group); location: home
Bernini et al [42]	Mixed methods	Touch-screen laptop or tablet	CBT^d^	Female: 5; male: 5; location: home
Bernini et al [43]	Quantitative	PC	CBT	Female: 17 telehealth and 13 in-person; male: 14 telehealth and 12 in-person; location: clinic (PC) or home (laptop)
Bogza et al [44]	Mixed methods	Web solution	Decision aid	Female: 6; male: 6; location: clinic, research center, or participant home
Bouzida et al [45]	Mixed methods	Robot with integrated touch screen	CBT	Female: 1; male: 2; location: home
Chang et al [46]	Quantitative	Screen and gesture sensitive device	Physical and cognitive training	Female: 8; male: 7; location: clinic
Chen et al [47]	Mixed methods	Touch-screen laptop or tablet	CBT	Female: 44 in evaluation group and 4 in focus group (same people); male: 13 in evaluation and 0 in focus group; location: clinic
Christiansen et al [48]	Qualitative	No device	Data collection (eg, UI^e^ design feedback, technology use or acceptance, icon design preferences, changes to cognition, cognitive training or games design, and needs for robotic assistants)	Female: 6 (2 were 70‐75 years and 4 were older than 81 years); male: 12 (4 were 70‐75 years, 6 were 76‐80 years, and 2 were older than 81 years); location: 15 at home and 3 in lab
Collette et al [49]	Qualitative	Integrated computer-based assistant	Health or medication management eg, BP^f^, reminders to drink water	Female: 7; male: 3; location: clinic
Contreras-Somoza et al [50]	Qualitative	Touch-screen laptop or tablet	Lifestyle (ADL) support, social interaction, or companionship	Female: 11; male: 2; location: clinic
Contreras-Somoza et al [51]	Qualitative	Touch-screen laptop or tablet	Lifestyle (ADL) support, social interaction, or companionship	Female: 21; male: 14; survey: 35 participants (15 Spain, 4 Netherlands, 2 Italy, 2 France, 4 Israel, 5 Serbia, and 3 Slovenia)
Cunnah et al [52]	Qualitative	Web solution	Social interaction, messaging, chat, or information provision	Total: 100; location: home-100 dyads (49 control, 51 intervention); intervention group got 1:1 training followed by group training; 25% drop-out; 75 dyads completed the study (39 control and 36 intervention).
Demiris et al [53]	Mixed methods	Smart conversational assistant with touch screen (eg, Google Home and Alexa)	Companionship and reminders (computer-based pet)	Female: 10; male: 0; location: home
Dixon et al [54]	Qualitative	No device	Data collection (eg, UI design feedback, about technology use or acceptance, icon design preferences, changes to cognition, cognitive training or games design, and needs for robotic assistants)	Female: 1; male: 1; location: home
Franco-Martín et al [55]	Qualitative	PC	CBT	Not stated
Gasteiger et al [56]	Mixed methods	Robot with integrated touch screen with pen and magnetic blocks (tactile sensors) Used 3 robots (Bomy 1, Bomy, and Silbot) in the project	CBT	Not stated; location: location most convenient to participants, including the university, workplaces, clinic, home, or via Skype (for experts; developed by Janus Friis, Niklas Zennström, Ahti Heinla, Priit Kasesalu, and Jaan Tallinn)
Gelonch et al [57]	Mixed methods	Wearable camera	Memory aid-digital camera records daily activities	Female: 4; male: 5; location: adult day center
Givon Schaham et al [58]	Mixed methods	Touch-screen laptop or tablet	CBT	Female: 13; male: 15; location: clinic and home
Guzman-Parra et al [59]	Quantitative	No device	Data collection (eg, UI design feedback, about technology use or acceptance, icon design preferences, changes to cognition, cognitive training, games design, and needs for robotic assistants)	Female: 576; male: 510; location: used secondary data; 1086 dyads: 299 with dementia, 787 with MCI; data not split for the MCI and dementia groups
Haesner et al [60]	Qualitative	No device	Data collection (eg, UI design feedback, about technology use or acceptance, icon design preferences, changes to cognition, cognitive training, games design, and needs for robotic assistants)	Female: 3; male: 3; location: clinic
Hassandra et al [61]	Mixed methods	VR headset and controller, hand motion trackers, or wireless mouse	Physical and cognitive training	Female: 19; male: 8; location: clinic
Heatwole and Kendra [62]	Qualitative	No device	Data collection (eg, UI design feedback, about technology use or acceptance, icon design preferences, changes to cognition, cognitive training, games design, needs for robotic assistants)	Female: 6; male: 4; location: outing location
Hedman et al [63]	Qualitative	No device	Data collection (eg, UI design feedback, about technology use or acceptance, icon design preferences, changes to cognition, cognitive training, games design, and needs for robotic assistants)	Female: 2; male: 4; location: home
Hedman et al [64]	Mixed methods	No device	Data collection (eg, UI design feedback, about technology use or acceptance, icon design preferences, changes to cognition, cognitive training, games design, and needs for robotic assistants)	Female: 18; male: 19; location: home; only 21 participants left at year 5
Horn et al [65]	Qualitative	Mobile phone and smartwatch	Memory aid-facial recognition aid to identify people	Female: 6; male: 14; location: home
Hu et al [66]	Mixed methods	No device	Data collection (eg, UI design feedback, about technology use or acceptance, icon design preferences, changes to cognition, cognitive training, games design, and needs for robotic assistants)	Female: 18; male: 13; location: clinic
Infarinato et al [67]	Mixed methods	A robot and a separate touch screen	Physical and cognitive training	Female: 8; male: 7; location: home
Irazoki et al [68]	Qualitative	Touch screen laptop or tablet	Cognitive training or cognition assessment	Female: 11; male: 2; location: clinic
Korchut et al [69]	Mixed methods	No device	Data collection (eg, UI design feedback, about technology use or acceptance, icon design preferences, changes to cognition, cognitive training, games design, and needs for robotic assistants)	Female: 36; male: 21; survey
Kubota et al [70]	Qualitative	Robot with integrated touch screen	CBT	Female: 0; male: 3; location: online session
LaMonica et al [71]	Quantitative	No device	Data collection (eg, UI design feedback, about technology use or acceptance, icon design preferences, changes to cognition, cognitive training, games design, and needs for robotic assistants)	Female: 127; male: 94; survey: 137 had MCI, 61 had SCI^g^, and 23 had dementia
Law et al [72]	Mixed methods	Robot with integrated touch screen	CBT	Female: 6; male: 4; location: lab
Lazarou et al [73]	Mixed methods	No device	Data collection (eg, UI design feedback, about technology use or acceptance, icon design preferences, changes to cognition, cognitive training, games design, and needs for robotic assistants)	Female: 9; male: 6; survey
Leese et al [74]	Mixed methods	Fitness tracker smartwatch	Collect daily movement and exercise data	Female: 1; male: 14; location: home
Lindqvist et al [75]	Qualitative	No device	Data collection (eg, UI design feedback, about technology use or acceptance, icon design preferences, changes to cognition, cognitive training, games design, and needs for robotic assistants)	Total: 5; location: clinic
Madjaroff and Mentis [76]	Qualitative	No device	Data collection (eg, UI design feedback, about technology use or acceptance, icon design preferences, changes to cognition, cognitive training, games design, and needs for robotic assistants)	Female: 3; male: 2; location: clinic
Maier et al [77]	Qualitative	Wearable watch, smartphone, and smartboard	Memory aid for daily activities or cognitive support or rehabilitation or share health information	Total: 6; location: clinic; 3 in focus group, 3 in usability evaluation of 2 prototypes; prototype 1 used a smartphone (not wearable technology) and prototype 2 used a smartphone and a smartboard (wall calendar)
Manca et al [78]	Mixed methods	Robot with integrated touch screen	CBT	Female: 9; male: 5; location: lab
Manera et al [79]	Quantitative	VR headset and controller or wireless mouse; screen and gesture-sensitive device; 3D glasses	CBT-train selective and sustained attention using image-based rendered environment	Female: 13; male: 15; location: lab
Mathur et al [80]	Mixed methods	Smart conversational assistant with touch screen (eg, Google Home and Alexa)	Health or medication management (eg, BP) reminders to drink water	Female: 4 total (phase 1‐2, phase 2‐2); male: 8 total (phase 1‐5, phase 2‐3); location: home
Matsangidou et al [81]	Mixed methods	VR headset	Regulate emotions and mood	Female: 19; male: 11; location: clinic
Mattos et al [82]	Mixed methods	PC and smartwatch	Insomnia intervention or sleep management	Female: 7; male: 5; location: home-only 10 completed the study
McCarron et al [83]	Mixed methods	Mobile phone and smartwatch	Memory aid-facial recognition aid to identify people	Female: 25; male: 23; location: home-29 participants had dementia
Mehrabian et al [84]	Qualitative	Touch screen laptop or tablet	Lifestyle (ADL) support, social interaction or companionship	Female: 19; male: 11; location: lab
Mondellini et al [85]	Mixed methods	VR headset and controller or hand motion trackers or wireless mouse	Cognitive training or cognition assessment	Female: 14; male: 1; location: lab
Moro et al [86]	Mixed methods	Humanoid robot, cartoon robot, and tablet	Lifestyle (ADL) support	Female: 6; male: 0; location: kitchen
Mrakic-Sposta et al [87]	Mixed methods	VR and stationary bike with controller on handlebars	Physical and cognitive training	Female: 6 (3 in experimental group, 3 in control group); male: 4 (2 in experimental group and 2 in control group); location: lab
Nie et al [88]	Mixed methods	Web solution and Webcam	Social interaction or messaging or chat or information provision	Total: 7 (5 in phase 1 and 2 in phase 2); location: clinic
Nieto-Vieites et al [89]	Mixed methods	Touch screen laptop or tablet	CBT	Female: 5 (study 2) and unknown (study 3); male: 3 (study 2) and unknown (study 3); location: clinic
Ortega Morán et al [90]	Qualitative	Touch screen laptop or tablet	CBT	Female: 16; male: 3; location: clinic
Park et al [91]	Quantitative	Touch screen tablet and wearable motion sensor device	Physical and cognitive training	Female: 12; male: 2; location: home
Park et al [92]	Quantitative	VR headset and controller or hand motion trackers or wireless mouse	CBT	Female: 7 control group and 7 VR group; male: 3 control group and 4 VR group; location: clinic
Piasek et al [93]	Mixed methods	Robot with integrated touch screen	Physical and cognitive training	Female: 3; male: 1; location: home
Pino et al [94]	Mixed methods	Robot with integrated touch screen	Lifestyle (ADL) support, social interaction or companionship	Female: 6; male: 4; location: clinic
Quintana et al [95]	Mixed methods	Touch screen laptop or tablet with tablet pen	Memory aid for daily activities, cognitive support, rehabilitation, or share health information	Female: 3 (Sweden) and 5 (Spain); male: Sweden-6 (Sweden) and Spain-5; location: clinic and home
Rossi et al [96]	Mixed methods	Robot with integrated touch screen	Lifestyle (ADL) support	Female: 2 (phase 1) and phase 3; male: 2 (phase 1) and phase 4; location: lab (phase 1) and home (phase 2)-
Saini et al [97]	Mixed methods	Videoconferencing (Zoom) and webcam	CBT	Total: 12; survey: 6 in each randomized group (face-to-face CBT or CBT via videoconferencing)
Scase et al [98]	Qualitative	Touch screen laptop or tablet	CBT	Female: development: 3 focus groups (group 1: 4; group 2: 4; group 3: 3), evaluation: 22; male: development: 3 focus groups (group 1: 5; group 2: 1; group 3: 1), evaluation: 3; location: clinic and home
Scheibe et al [99]	Qualitative	TV with remote control or tablet; sphygmomanometer (to measure blood pressure)	Telemonitoring medical app to communicate with multidisciplined health professionals	Female: 8; male: 4; location: home
Shamir et al [100]	Mixed methods	Touch screen laptop or tablet	CBT	Female: 6; male: 8; location: home
Shellington et al [101]	Mixed methods	Mobile phone	CBT	Female: 14; male: 5; location: home
Shin et al [102]	Qualitative	Remote controlled robot (robot is not with user, but controlled via app on PC, tablet, or mobile phone)	Lifestyle (ADL) support	Female: 0; male: 6; location: clinic or home
Stogl et al [103]	Quantitative	Robotic walker (user holds handles and pushes the device for exercise)	Physical and cognitive training	Female: 2; male: 8; location: clinic
Stramba-Badiale et al [104]	Mixed methods	VR (or TV), joystick, and foot pad or rudder	Assist with navigation and training of spatial memory	Female: 4; male: 3; location: lab
Tuena et al [105]	Mixed methods	VR (or TV), joystick, foot pad or rudder, and 3D glasses	Assist with navigation and training of spatial memory	Female: 2; male: 6; location: lab
Van Assche et al [106]	Qualitative	Robot with integrated touch screen	Data collection and design guidance for robot use cases	Female: 16; male: 14; location: home
Votis et al [107]	Qualitative	Touch screen laptop or tablet	CBT	Total: 17; location: lab
Wargnier et al [108]	Mixed methods	Smart conversational assistant with touch screen (eg, Google Home and Alexa)	Health or medication management (eg, BP and reminders to drink water)	Female: 11; male: 3; location: clinic (5 had Alzheimer disease)
Weering et al [109]	Qualitative	Web solution	Physical and cognitive training	Female: 43; male: 14; location: clinic
Wolf et al [110]	Mixed methods	VR headset	Lifestyle (ADL) support	Female: 6; male: 0; location: clinic kitchen
Wu et al [111]	Qualitative	No device	Data collection (eg, UI design feedback, about technology use or acceptance, icon design preferences, changes to cognition, cognitive training, games design, and needs for robotic assistants)	Female: focus group 4, interview 12; Male: focus group 1, interview 3; location: lab
Wu et al [112]	Mixed methods	Robot with integrated touch screen	Lifestyle (ADL) support	Total: 6; location: clinic
Yamazaki et al [113]	Qualitative	Robot (sitting on table, programmed to interact with user, sing, talk, and respond to spoken words)	Companionship	Female: 2; male: 0; location: home
Yun et al [114]	Quantitative	VR headset and controller or hand motion trackers or wireless mouse	CBT	Female: 6; male: 5; location: clinic
Yurkewich et al [115]	Mixed methods	Touch screen laptop or tablet	Social interaction, messaging, video chat, or information provision	Female: 6; male: 2; location: home
Zafeiridi et al [116]	Mixed methods	Web solution	Lifestyle (ADL) support, social interaction, or companionship	Female: 14; male: 10; location: home
Zedda et al [117]	Mixed methods	Robot with integrated touch screen	CBT	Female: 6; male: 10; location: clinic
Zhang and Gao [118]	Mixed methods	No device	Data collection (eg, UI design feedback, about technology use or acceptance, icon design preferences, changes to cognition, cognitive training, games design, and needs for robotic assistants)	Total: 5; location: clinic
Zhang and Liu [119]	Mixed methods	Touch screen laptop or tablet with foot stand with pressure sensors	Physical and cognitive training	Female: 79; male: 58; location: survey and usability evaluation in lab: 137 responses for phase 1 (requirements gathering) but only 30 responses were from people with MCI; 107 responses were from family of people with MCI
Zhu et al [120]	Mixed methods	Mobile phone	Memory aid-digital storytelling	Female: 9; male: 3; location: clinic
Zubatiy et al [121]	Mixed methods	Smart conversational assistant with touch screen (eg, Google Home and Alexa)	Lifestyle (ADL) support	Female: 4; male: 6; location: home (MCI and caregiver dyads included in study)

a VR: virtual reality.

b MCI: mild cognitive impairment.

c ADL: activity of daily living.

d CBT: cognitive behavioral therapy.

e UI: user interface.

f BP: blood pressure.

g SCI: subjective cognitive impairment.

Below, we first list 4 broad categorizations of the examined studies in terms of (1) study details and nature of participants, (2) purpose of technological solutions, (3) types of interaction devices, and (4) interaction modalities, and provide a short description of how studies evaluated usability. This is followed by the resulting themes derived from analysis of participant feedback.

### Categorization of Examined Study Details

#### Characteristics of Included Studies and Study Participants

Over half of the included studies (n=45) were conducted in Europe, with almost a quarter (n=10) of these in Italy. Some of the European studies included participants from multiple countries [51,59,67,69,90,95,116]. Table 1 shows that 61 studies included fewer than 20 participants, 49 studies included fewer than 15 participants, 25 studies included fewer than 10 participants, and 8 studies included fewer than 5 participants. Many studies commented on the lack of cultural diversity among their participants.

Studies used a mixed methods (n=47), qualitative (n=26), or quantitative (n=10) approach. Studies that did not explicitly declare their design type are classified in this paper based on the type of data collected. Qualitative studies used semistructured interviews, focus groups, workshops, and observations to collect data. Quantitative studies used surveys or questionnaires with closed questions to gather data. Most studies, regardless of type, collected quantitative data about participant demographics. Quantitative data about usability, acceptability, and user experience (UX) were typically gathered using questionnaires with responses on a Likert-type scale.

#### Purpose of Technology Solutions in Studies

Over a third (n=32) of solutions provide cognitive training in the form of cognitive behavior therapy (CBT; n=21), a combination of CBT and physical training (n=9), or CBT and cognitive assessment (n=2). Other functions mentioned as needs by participants and supported by solutions were lifestyle or activities of daily living support (n=13), memory training or management (n=6), health and medication management (n=4), staying in touch with family and friends, social engagement, entertainment (n=4), companionship (n=2), spatial navigation or orientation (n=2), insomnia intervention (n=1), and daily exercise monitoring (n=1). Solutions also provided support for decision support (n=1) and mood regulation (n=1), but these were not identified as needs by participants. Further needs identified by participants but not supported by solutions were the management of finances and help to locate lost items. Sixteen studies were designed to collect data to (1) guide the design of future solutions, (2) inform amendments for existing solutions, or (3) collect information about the needs of people with MCI.

#### Interaction Devices

Studies included various technology platforms and devices as presented in Table 2 in the Multimedia Appendix 1. Almost half (n=37) of the proposed solutions include a touch screen as a component of a laptop or tablet (n=17), a robot (n=14), or a mobile phone (n=6). This reflects research by [25,122], which showed that touch screen devices are effective and easy to use by older adults. Some (n=4) robot solutions did not include sound, but the majority (n=12) included voice or sound. Eleven solutions involved the use of virtual reality (VR). Eight solutions included wearables (other than VR headsets or 3D glasses) in the form of a smartwatch (n=5), a wearable motion sensor (n=1), a sphygmomanometer to measure blood pressure (n=1), and a wearable camera (n=1). Six studies included a mobile phone, among which 3 paired the phone with a smartwatch. Five studies used a conversational agent such as Google Home (n=3), an integrated conversational agent on a laptop (n=1), and a computer-based pet avatar on a laptop or tablet (n=1).

#### Interaction Modalities

The variety of technology and devices made different options available for users to interact with the solutions and vice versa. Over half the studies (n=48) involved touch interaction either as unimodal (n=24) or multimodal (n=24). Seven of these touch solutions could also be used with a keyboard and mouse. This reflects research by [25,122], which demonstrates that touch screen interaction is an effective interaction mode for both old and young users, even with some usability issues. Nine studies combined a touch screen device with speech output, while 13 studies combined a touch screen device with speech input and output. Combining visual and speech modalities is supported by research findings that multimodal interaction is more effective and usable for older adults [123], as compared to unimodal touch or speech [124]. Voice or sound was associated with 29 solutions; 13 studies involved solutions that provided speech or sound outputs, and 16 studies involved solutions that afforded speech or sound input and output. Voice or sound outputs were associated with VR solutions (n=4), robots (n=6), tablet device solutions (n=2), and a web solution (n=1). Voice or sound input-plus-output was associated with VR solutions (n=2), robots (n=6), tablet device solutions (n=3), embodied conversational agent solutions (n=3), a web solution (n=1), and a mobile phone app (n=1). Only 2 studies used gesture as an interaction modality; 1 used a robot that gestured to the user as a demonstration of extraversion [117], and the other was a cognitive game based on Tetris, which included a user gesture capture device [46]. Four studies used haptic (vibration) interaction via a smartwatch and a touch screen device (n=2), a vibrating keyboard with a touch screen device (n=1), or a vibrating handset as part of a VR solution (n=1). Additional interaction modalities provided by VR solutions included joysticks and pedals or rudders (n=4), and robot solutions with lights, facial expressions, and movement (n=7). Table 2 in the Multimedia Appendix 1 lists the device and interaction modality combinations for each study.

#### How Studies Evaluated Usability or Acceptability

Studies used different methods to evaluate usability. Some studies used the System Usability Scale [42,44-46,61,80,81,88,95,104,105,109], which is a simple, 10-item Likert scale used to assess usability [125]. Research [126] has shown that the System Usability Scale is suitable for evaluating digital health applications. Some studies of robotic solutions used the Almere questionnaire [127], which is based on UTAUT and developed to evaluate acceptance of assistive social agents by older adults [86,93,94,96,108,112]. Several studies designed their own questionnaires based on TAM, TAM3, STAM, MATOA, or UTAUT.

### Themes Derived From Thematic Analysis of Participant Feedback

Inductive thematic analysis of extracted data identified five themes: (1) purpose and need, (2) solution design and ease of use, (3) self-impression (how the solution makes the user feel), (4) lifestyle (how well the solution fits with the user’s life and routines), and (5) interaction modality.

#### Purpose and Need

Participants identified that improving or maintaining their existing cognition level is the primary need, and over a third of solutions focused on this function. The remaining studies reveal other needs identified by participants, and only 2 studies provided solutions for activities that were not identified as needs. Assistance with managing personal finances was identified as a need [75,76]; however, none of the studies provided a solution to this end. Being able to manage one’s own finances decreases vulnerability and is an important enabler for independence. Further investigation is needed to determine how to support older adults with MCI to have control of and manage their finances in a safe and secure manner. Participants also identified help to find lost items as a need [93], which was again not supported by any studies. The answer to RQ1A is that proposed solutions for older adults with MCI are perceived as useful, but there are also gaps where functional support is missing. Given the demonstrated importance of perceived usefulness for TA, this is an area where further work is needed.

#### Solution Design and Ease of Use

Study participants reported a variety of usability and usage issues and noted that an ability to configure and personalize a solution provides flexibility and increases usability.

*There’s all grades of dementia as well isn’t there. And some would need more help than others*.

[56]

Participants said it would be useful if they could adjust (1) solution tasks to match user skills [42,49,52,60], (2) task complexity [45,55,56,81], (3) task workflow [115], (4) timing of reminders [65], (5) user interface design (colors of background, text, and widgets [56,98], size of text and widgets [47,66], and color contrast [56]), (6) speed, volume, and accent of speech [56,95], (7) language (formal or casual) [108], (8) robotic facial features and height [69,86,94,112], and (9) avatar image [50].

Consistent design, labeling, and widget placement were mentioned as important enablers of usability [88,120], and this is especially critical for users with cognitive impairments or memory concerns. Participants added that they may use multiple toolsets and that seamless integration and consistency across these toolsets would increase usefulness and ease of use.

Participants provided ideas about how to increase usability of solutions, such as (1) reduce or eliminate the need to remember passwords or specific trigger words because this increases complexity [121], (2) make messages specific and include sufficient detail so the message is actionable [80], (3) provide sufficient time to read text messages [42,68,105], (4) use language appropriate for older users [98], (5) use simple, familiar, and common words [88,95], (6) use sound as well as visual indicators to make it obvious that an action was performed successfully [95], (7) avoid loud noises and too many animations because these create a distraction [60], and (8) make task steps simple [119]. Participants prefer solutions that provide immediate feedback about task progress and task completion because they are engaging and provide motivation and incentives for use [60,63,74,80,90,64]. This illustrates how UX can influence TA.

Specific feedback points about user interfaces were (1) layout should be simple and not too busy or overwhelming so that controls are obvious and the interface does not overstimulate or place a burden on the user’s cognition [44,68,88,120], (2) use visual aids [70], (3) use simple images so the item is easily recognizable [66,110], (4) use intuitive and simple navigation [41,107], (5) use bright and contrasting colors [89,98,107], (6) use large text, buttons, and widgets [40,88,107], (7) ensure all options are visible so information is transparent [44], and (8) list options in order of user priorities because users often select the first option listed [44]. These comments are consistent with design principles proposed by Nielsen [128-130].

Reliable technology creates trust, confidence, and feelings of empowerment [94,95]. Studies where participants experienced technical issues [56,67,72,108], or if the device had a low battery life or charged slowly [99], resulted in frustration and negative feedback.

Participants commented that age-related physical limitations may affect how they use technology, so accessibility is an important design feature of technology solutions for older adults with MCI [40,95,98,121]. Context of use and location were also raised as considerations in this regard (eg, rural vs metropolitan and speed of internet connection [55]).

Studies showed that older adults with MCI prioritize security and privacy [48,94]. Some participants said they prefer robots to have mechanical features and to be shorter than a person [94,112] because they felt less under surveillance. This preference has also been reported by other research [131]. Safety was identified as a priority, related to both (1) personal safety when using technology (especially robots or VR solutions) and (2) when using the internet or social media [48]. Participants said that even though privacy is a need, they are willing to compromise privacy and share personal data or their location with medical professionals or carers in an emergency [73]. Some participants said that it would be useful to receive regular reminders of what data is being shared [116].

Given the breadth of usability issues reported and the number of changes suggested, the answer to RQ1B is that proposed solutions for older adults with MCI are not perceived as completely easy to use, and this is an area where future technologies need to focus.

#### Self-Impression

Participants commented that the feelings they experience while using a solution can positively or negatively affect their comfort, confidence, and overall well-being. The wearable camera solution made participants feel uncomfortable because it drew attention to them, and they felt conspicuous.

*If people don’t know you, they look at you as if they are thinking. Well, what is he doing?*

[57]

Participants also felt vulnerable because the camera may capture private moments, such as when they are going to the bathroom.

*The camera is black and people look at it a lot. I think that if it were a more natural color, people would look at it less*.

[57]

Some participants said that technology would be useful for other people, but not themselves. Technology is often associated with negative aspects of aging, such as loneliness or ill health [111] because it advertises the user’s limitations and lack of independence.

*The telecare system would be useful for me if I had more deficits. But so far, I can manage by myself at home*

[84]

Participants said they want solutions to reflect their identity and present a positive self-image. They suggested that solutions should (1) use affirming language that does not remind them about their cognitive limitations [40,68,72,76], (2) include nondirective task workflows (eg, participants prefer a system to check in rather than remind [80]), and (3) not draw attention to the user or the solution; this is especially true for robotic solutions [94,111,112] and the wearable camera [57]. Self-concept and social presence have been shown to be determinants of TA in the MATOA model, and a literature review [132] describes similar feedback.

Participant personality traits did not appear to affect acceptance of robots in the long term [96]; however, differences were observed over the study duration. Some participants felt comfortable engaging with a robot and interacted with it in a human-like manner [86,106]. They said a robot is novel and suggested that it should be human-like with autonomy, be able to perform simple tasks, and interact via 2-way communication [45,69]. This is consistent with existing research [133], which demonstrated that robots that take initiative, exhibit more activity, and are extraverted are better accepted by users.

*I don’t see much difference between [my doctors] and the robot except one is ran by electricity and the other one is a human being.*

[45]

Other participants said they want to engage with real people rather than anthropomorphic technology. They value human connection [88,106,111], and there is a concern that the use of technology will reduce their existing human contact. These participants preferred more machine-like robots that did not imitate human traits.

*A robot doesn’t have a heart, It must be for people who are very handicapped. It’s not for me*. *It makes me think that my life is terminated. I’d rather die than live with a robot.*

[112]

Other concerns identified by participants relating to the use of robot solutions were that the robot would (1) be hard to use or experience technical problems, (2) not do what they needed or wanted it to do, (3) be expensive, (4) increase isolation, or (5) make them too dependent on the robot [94,111,112]. Nevertheless, many participants felt optimistic about anthropomorphic solutions and recognized their potential.

*The human aspect, the “feeling,” is hard to create with a robot. But there is an enormous progress in that world, and I do believe in it. Of course, still in combination with us, humans.*

[106]

Participants who said they prefer more machine-like robots were less willing to create an emotional connection with the robot [111,112]. However, some participants got very attached to their robot solutions, treated the robot like a friend, and became sad when the study ended [53,69,78,113]. This raises important issues for designers about how to support users if technology solutions fail or become unavailable.

#### Lifestyle

Participants said that independence and autonomy are important, and some said they felt a responsibility to their relatives and carers to maintain their self-sufficiency [45]. Participants want to feel in control of their lives [45,51,60,75,76,80,88,103,111], and technology solutions that create a sense of user empowerment are preferred rather than solutions that impose mandatory interventions or training [60]. While participants appreciate the increased independence technology can provide [76], they are also concerned about receiving too much assistance (from technology or people) in case they become reliant on this and lose their autonomy [111] or independence [69,76]. The studies reinforced that IT literacy, training, and availability of support from carers and family are enablers for acceptance and continuing use of technology [47,48,63], which is consistent with TAMs. However, it is important that technology solutions do not create a dependence on support services, which in turn would erode independence.

Cost was identified by study participants as an important factor when considering adoption of a technology solution [63,84,94,106,112]. This is consistent with UTAUT2 and H-TAM, which include cost, price, and value or perceived benefit as determinants of adoption.

Physical comfort and convenience are important considerations for the use of technology. Most participants enjoyed the novelty of VR solutions; however, some experienced visual discomfort or nausea, found the headsets uncomfortable, or said the VR solutions were not practical [79,81,110, 39].

*Well, I liked it, but it was too heavy and big for me. I had difficulty breathing because it wasn’t staying in place and fell into my face blocking my nose*.

[81]

The answer to RQ2A is that participants prefer devices that are light and portable (mobile phone or tablet), commonly owned (mobile phone), readily available, and have a reasonably large screen [74,84,119,120]. This review also identified that older adults with MCI do not like small wearable technology, such as watches, because the screens are too small, and many participants said that watches are not convenient because they are no longer part of their everyday toolset [74]. In addition, the physical size of the technology solution and its placement in the home affect ease of access and contribute to convenience (eg, smaller robots take up less room and are less intrusive in a home setting [45,106]).

Studies where solutions were integrated into participants’ existing lifestyles and routines made participants feel comfortable, confident, and more in control. This is consistent with the STAM, MATOA, and H-TAM models, which show that compatibility, ease of use, and maintaining a sense of control are important for older users of technology.

*I like that Google kind of feels like a part of the house, and not something that I have to keep answering to all the time like my morning alarm*.

[80]

#### Interaction Modality

Study participants provided positive feedback for voice interaction and said it was intuitive and easier than writing [45]. They also mentioned challenges such as (1) the solution’s accent was hard to understand or the solution did not understand the user’s accent or speech pattern [56,72], (2) the speech was too fast or the volume was too loud or too soft [68,78], (3) the solution spoke for too long [108], (4) the solution only recognized a limited vocabulary and participants had to remember specific “trigger” words to activate the solution or give it commands [53,110,121].

*If it was more New Zealand English spoken it would be easier for me I think*.

[56]

Similar feedback has been documented by other researchers studying voice interaction with older users [27,32]. Research has demonstrated that subtle differences in speech can be observed in older adults with MCI [30], and people with cognitive decline speak slower, in smaller chunks, pause more often, have longer silences, respond slower to questions, and provide shorter answers [134]. This highlights the importance of including older adults with cognitive impairments when designing and evaluating voice interaction solutions. Mozilla [135] is leading the world’s largest open-source voice data project called “Common Voice.” The project is building and refining a voice dataset that is being used to teach machines to speak, and so far, it includes 131 languages and 32,584 hours of recorded voice data. However, analysis [136] shows that people older than the age of 90 years are represented only in the Mongolian and Polish languages, and people in their 80s are only represented in the Esperanto, Turkish, Abkhazian, Toki Pona, and Hebrew languages. Of the English-contributing voices, there are 0.01% in their 70s, 0.04% in their 60s, 0.05% in their 50s, 0.09% in their 40s, 0.14% in their 30s, 0.25% in their 20s, 0.06% in their teens, and 0.36% of unspecified age. It is not known if any of the recorded voices are from older adults with MCI. Technology solutions using speech interaction increase their risk of error if they cannot recognize the speech of older users or the speech of people who are cognitively impaired. Users’ current optimism creates an opportunity to invest in voice interaction to improve its usability and effectiveness.

Participants said that touch interaction is effective and easy, especially for less IT literate people [55,107], but they experience difficulties such as (1) trouble dragging items across the screen, (2) knowing how much pressure to use [100], (3) small screen or widget size making it hard to select the target accurately, and (4) using the touch screen if the user has dexterity problems [41,72,95,100]. Some participants chose to use an instrument to avoid these challenges [41,95].

*I have a problem only with, first, with the gentleness of the finger. My finger isn’t the gentlest thing*.

[100]

These findings are consistent with outcomes from a literature review investigating the use of touch screens by older users [122], and an investigation of touch interaction, which identified usability issues associated with clicking, dragging, zooming, and rotating on a touch device for both older and younger users [25]. Based on feedback from study participants in this review, it appears that these challenges have not been fully resolved.

Participants said robotic assistants should be able to engage in both a verbal and nonverbal manner, and they preferred robots that can speak, listen, and answer questions rather than those with 1-way interaction via a touch screen [45,69]. If the solution is embodied, they want to be able to touch it physically and interact with it rather than simply view it on a screen [53]. Active robots that performed tasks were deemed more pleasant [96]. This is consistent with findings that robots with unimodal auditory 2-way feedback are highly usable and acceptable for older adults [27].

Participants were agnostic to haptic interaction, except for conveying that a smartwatch was not a preferred device, and suggested that it is useful to be able to control technology remotely [108,119], which supports the need for comfort and convenience.

The answer to RQ2B is that participants prefer multimodal interaction, in particular speech, visual or text, and touch [60,80,88,115,118,121] because it is flexible, provides options for use if people have physical or cognitive limitations, and makes solutions accessible to people with poor hearing or vision or speech limitations. This supports existing research about the effectiveness of different interaction modalities for older users, which showed that multimodal interaction is most effective [28,29,137] and that the effectiveness of interaction modalities and multimodalities is task- and user-dependent [26,29,138]. However, no studies included older adults with cognitive impairment, which creates a gap in understanding the effectiveness of different interaction modality combinations for this population.

## Discussion

### Principal Findings

Participants across studies reported similar needs but often in a different priority order, and this scoping review cannot (1) provide a definitive priority order for needs; (2) identify if needs are relatable to technologies or devices; or (3) attribute differences to the context of use, cultural differences, participant demographics, or the way MCI is affecting participants’ lives. Mondellini et al [85] found that user needs appear to be location-specific, but further research is required to understand which needs are location-specific versus those that are generalized.

Fadzil et al [139] completed a review of existing technology solutions for older adults with cognitive impairments and found that current solutions are focused on (1) caregiver support, (2) strengthening cognition, (3) cognitive rehabilitation, and (4) using technology for disease prevention or self-management. Fadzil et al [139] do not comment on reported needs, technology preferences, or opinions about existing solutions for older adults with cognitive impairments. Neither does it describe how well existing technology supports user needs, nor how well technology is aligned with users’ preferred ways of doing things. This gap contributes to the lack of understanding of TA by older adults with MCI.

Our review identified that security and privacy are important for older adults with MCI; nonetheless, they are prepared to compromise these to feel safe. However, they do not want to be continuously watched or feel like they are under surveillance. Nastjuk et al [140] used the term “techno-invasion” to describe the stress caused by constant observation and monitoring. Solutions must balance the need for users’ privacy and security with their need for safety and well-being.

A positive UX is a significant enabler for technology acceptance, and ease of use contributes greatly to UX, which suggests that designers must give both priority. User attitude has been identified in TAMs as a determinant of use, and several studies commented that MCI has an impact on a person’s attitude toward technology use [48,74]. This observation has also been made by other researchers [43], who noted that we need a greater understanding of how sociodemographic factors and cognition affect attitude.

Some older adults with MCI are not willing to connect with a robot because they prefer to interact with a human, while others are comfortable engaging with anthropomorphic solutions. Our review identified that participants who created an emotional connection with an anthropomorphic solution were vulnerable to becoming depressed once the solution was no longer available. This raises important issues for designers about how to support users if technology solutions fail, and ethical issues about how to manage emotional connection and attachment to more anthropomorphic solutions. It illustrates that technology represents part of the solution but not the entire solution [75], and that service providers must balance the need for human connection with the need to increase efficiency and reduce demand on health services.

Our review found that participants experience a variety of usability and usage issues, and that the flexibility of use and the ability to configure a solution increase ease of use. Older adults with MCI feel more comfortable, confident, and in control when solutions are consistent, predictable, and integrate into their existing lifestyles and routines. People often use multiple toolsets, and technology providers should prioritize consistency and seamless integration of their products. Overall, our analysis reveals that technology solutions should support how people behave and live, not require them to change and adapt to how the technology operates.

Some studies recommend that technology solutions for older adults with MCI should be designed together with this population [48,73,74,95,102,107,116] to ensure that all needs are identified and appropriately prioritized. This recommendation is supported by our findings, which show that while there is consistency in the top-priority need, there are differences in how participants in different locations prioritize the order of needs. Existing research shows that there is cultural and contextual influence on TA, and differences in the needs and priorities for independent living of older adults in Sweden and Poland, and the technologies they would consider using [141]. Given the evidence for the importance of perceived usefulness and ease of use as factors for TA, it is critical to understand the needs and characteristics of the target user base so we can design to maximize TA. User-centered design (UCD), first proposed by Kling [142], is a design methodology that focuses on usability and UX. UCD promotes the inclusion of end users in every stage of solution creation, starting with initial data gathering to understand users’ characteristics, environment of use, and requirements. UCD is iterative and incorporates regular artifact evaluation and refinement based on user feedback. UCD processes and techniques have been applied successfully to design assistive technologies for people with dementia and shown to be an effective way to accurately capture requirements and ensure solution usability and acceptance [143].

### Scope of Future Work

This review revealed that older adults with MCI have opinions about how and when they want to use technology, and it is imperative that future solutions are designed collaboratively with this population. Research is needed to accurately identify the needs of older adults with MCI so we understand what would be considered useful and how best to position technology in their lives. It would also be beneficial to identify needs that are common and which needs vary depending on demographics, location, or other factors.

Solutions designed for older adults with MCI must generate a positive experience because this affects a user’s comfort, confidence, and overall well-being. This review collated suggestions from participants, but more research is required to fully understand the factors to be considered.

Participant feedback suggests that personalization and control, and enablement are important for older adults with MCI, but it is not clear how choice with respect to technology, device, and interaction modality affects TA. This review identified that older adults with MCI do have preferred devices and interaction modalities; however, further work is needed to fully understand the effectiveness of different combinations of interaction modalities and how these are influenced by the effects of MCI, context of use, device being used, and task being performed.

Based on the findings, older adults with MCI want to have autonomy, independence, and feel safe. They are accepting of technology if it positively contributes to their lifestyle and provides a sense of well-being. Future research should aim to provide clarity about how to effectively meet safety, privacy, and security needs and establish technology as an enabler for independence and autonomy in older age.

Finally, the collection of longitudinal data about technology use by older adults with MCI would capture changes to usage patterns, preferences, and adoption factors as MCI advances and enable us to understand how solution designs should allow for adaptation to support MCI progression.

### Study Strengths and Limitations

This review comprehensively searched 9 databases for studies over a 10-year period. It considered research about a broad set of technology solutions proposed for older adults with MCI in the pre– and post–COVID-19 environments.

However, the study is not without limitations. This scoping review only included studies in English, so it is possible that untranslated relevant studies were overlooked. Most studies published their outcomes promptly after data collection; however, [56] began data gathering in 2016 but did not publish the results until 2022. This is a long time in terms of technology evolution and potential for change in people’s opinions and attitudes. Some studies did not analyze nonverbal feedback, so it is possible that important information was missed [50,68].

Most studies established an MCI diagnosis using the Montreal Cognitive Assessment [144], the Mini-Mental State Examination [145], or Petersen’s criteria [146]. However, the cutoff criteria for each assessment tool were not applied consistently across studies.

The definition of “older adult” is not consistent across the 83 studies. Most studies considered older than 65 years as “older;” however, some studies included participants aged between 48‐59 years. Some studies did not have an equal representation of genders.

Many studies listed small participant numbers or a lack of cultural diversity as limitations, and most studies included participants from only one location, meaning it may not be possible to generalize outcomes. Less than half of the studies were completed in a real-life setting, so outcomes may not be fully representative of real-life results.

Studies did not collect a consistent set of participant data (such as IT literacy, existing devices and technology being used, education level, or profession), so it is not possible to comment on how these may have impacted participants’ opinions, although age, sex, education level, or experience are listed as moderators in existing TAMs such as TAM, UTAUT, and MATOA.

Some studies did not have people with MCI represented in all phases or excluded people with physical limitations, so the opinions of these people have not been considered. In addition, the perspectives of other important stakeholders, such as medical and allied health professionals who treat older adults with MCI, are not considered in this paper. A study exploring the perspectives of 20 medical and allied health professionals from France, Italy, Japan, and Germany, who treat older adults with cognitive impairments [147], identified that physical, social, and cognitive interventions are considered important, and technology is an effective way to deliver and support these. This scoping literature review is the first part of a multistage research project, and future studies will include other stakeholders, such as medical and health professionals, family, and caregivers.

### Conclusions

This scoping review identified that improving or maintaining their current level of cognition is the top-priority need for older adults with MCI. Proposed technology solutions are perceived as useful by this population, even though gaps exist where functional support is missing. However, they are not perceived as completely easy to use, due to a variety of usability issues. In addition, technology solutions may have a positive or negative effect on the user’s comfort, confidence, self-esteem, self-image, and overall well-being, depending on the feelings and emotions triggered by the solution design. Older adults with MCI have preferences for how they use and interact with technology, and which devices they use. They prefer multimodal interaction—especially speech, visual or text, and touch—because it is effective and provides options for use, and devices that are comfortable, convenient, lightweight, readily available, cost-effective, and have large screens. Future work should focus on (1) gathering information about the needs of older adults with MCI and clarifying how these are dependent on location, demographics, or other factors; (2) improving ease of use and UX; (3) enabling customization and personalization; (4) further exploring interaction preferences and the effectiveness of different interaction modes; (5) providing options for multimodal interaction; and (6) integrating technology solutions more seamlessly into people’s existing lifestyle and routines.

## Supplementary material

10.2196/78229 Multimedia Appendix 1 Study extract data.

10.2196/78229 Checklist 1 PRISMA-ScR checklist.

## Abbreviations

CBT: cognitive behavioral therapy
H-TAM: healthcare technology acceptance model
HCI: human-computer interaction
MATOA: model for the adoption of technology by older adults
MCI: mild cognitive impairment
PRISMA-ScR: Preferred Reporting Items for Systematic Reviews and Meta-Analyses Extension for Scoping Reviews
STAM: senior technology acceptance model
TA: technology adoption
TAM: technology acceptance model
UCD: user-centered design
UTAUT: unified theory of acceptance and use of technology
UX: user experience
VR: virtual reality
